# Combined analysis of expression, prognosis and immune infiltration of GINS family genes in human sarcoma

**DOI:** 10.18632/aging.204191

**Published:** 2022-07-27

**Authors:** Kexin Zhang, Jian Zhou, Tong Wu, Qunyan Tian, Tang Liu, Wanchun Wang, Hua Zhong, Ziyuan Chen, Xungang Xiao, Gen Wu

**Affiliations:** 1 Department of Orthopedics, The Second Xiangya Hospital of Central South University, Changsha 410011, Hunan, China; 2 Department of Psychology, School of Public Health, Southern Medical University, Guangzhou 510515, China; 3 Department of Orthopedics, The Fifth Affiliated Hospital, Southern Medical University, Guangzhou 510900, Guangdong, China; 4 Department of Orthopedics, The First People’s Hospital of Changde City, Changde 415003, Hunan, China; 5 Department of Orthopedics, Chenzhou No.1 People’s Hospital, Chenzhou 423000, Hunan, China

**Keywords:** sarcoma, prognosis, expression level, GINS

## Abstract

Objective: This study was undertaken to explore the expression and prognostic value of GINS family in human sarcoma, as well as the association between the expression levels of the GINS family and sarcoma immune infiltration.

Results: We discovered that the mRNA expression levels of GINS1, GINS2, GINS3, and GINS4 were all higher in the majority of tumor tissues than in normal samples, of course, including sarcoma. Through the CCLE, all the four members expression were observed in high levels in sarcoma cell lines. In Gene Expression Profiling Analysis (GEPIA) and Kaplan-Meier Plotter, our results indicated that the poor overall survival (OS), disease-free survival (DFS) and relapse free survival (RFS) were tightly associated with the increased expression of GINS genes. In TIMER database, we found that highly expressed GINS was significantly correlated with the low infiltration level of CD4+ T cell and macrophage.

Conclusions: The four GINS family members were all the prognostic biomarkers for the prognosis of human sarcoma and can reduce the level of immune cell infiltration in the sarcoma microenvironment.

Methods: In terms of the expression levels of mRNA for GINS family members, a particular contrast in various cancers, especially human sarcoma, was conducted through ONCOMINE and GEPIA and CCLE databases. Kaplan-Meier Plotter was used to identify the prognostic value of GINS family in sarcoma. The relationship between the expression level of GINS and the infiltration of immune cells was analyzed in TIMER database.

## INTRODUCTION

Sarcomas consist of a group of rare solid tumors of mesenchymal cell origin with distinct clinical and pathological features. Generally, they are divided into two categories: soft tissue sarcoma and osteosarcoma. Sarcomas account for approximately 1% of all adult malignancies and 15% of pediatric malignancies [1]. Osteosarcoma accounts for 20% of primary malignant bone tumors, it is the most common primary malignant bone tumor in adolescents. 70%-80% of all the patients are 10-25 years old. The annual incidence rate is 1-3 people per 1 million people [2]. As the pathogenesis of osteosarcoma is still unclear, the 5-year survival rate of patients with osteosarcoma still hovers between 60% and 70% [3]. Soft tissue sarcomas (STS) (including fat, muscle, nerve and nerve sheath, blood vessel and other connective tissues) is classified according to similar tissue morphology. The STS classification reported by the World Health Organization in 2013 includes more than 50 histological subtypes, liposarcoma (LPS), and Differentiated sarcoma (US), leiomyosarcoma (LMS), myxofibrosarcoma (MFS) and synovial sarcoma (SS) are the most common types in adults [4]. Despite the development of surgery and adjuvant therapy, the treatment of sarcoma has improved [5], but the long-term survival rate of patients with high-grade sarcoma is still very low [6]. More than 50% of high-risk sarcoma patients will metastasize and die. Therefore, it is urgent to further explore the molecular mechanism of the occurrence and development of sarcoma [7].

CMG complex is composed of the Cdc45 protein, four subunits of the GINS complex (Psf1, Psf2, Psf3, and Sld5) and the heterohexamer minichromosome maintenance (MCM) 2-7 complex. It is most likely to be used as a replication helicase to unbind duplex DNA before the replication fork [8]. Recently, studies have shown that impaired function of the CMG complex will accelerate the development of genetic diseases [9]. The GINS complex consists of four subunits encoded by GINS1, GINS2, GINS3 and GINS4 genes. As a member of CMG, GINS complex plays important roles in the establishment of DNA replication forks and replisome progression. We knew that GINSs were highly expressed in many tumors and were associated with their prognosis. For example, GINS1 was found to be a potential biomarker for patients with CRC [10], and an inherited GINS1 deficiency was found to be one of the foundations of growth retardation [9]. GINS2 served as an independent risk factor for poor prognosis of early-stage cervical cancer through multivariate analysis [11]. Liu et al. found that GINS mRNA expression was significantly increased in patients with alcohol abuse. In experiments of mice, alcohol stimulated inflammatory cytokines and GINS2 knockdown further promoted them, while GINS2 knockdown promoted the alcohol-induced NF-κB activity of microglia, suggesting that GINS2 had a protective mechanism in alcohol-induced brain injury [12]. GINS3 has not only been linked to colon and lung cancer [13, 14], but also plays a central role in the eukaryotic replicative helicase CMG (Cdc45-MCM helicase-GINS) [15]. GINS4 has also been associated with the progression and prognosis of lung and stomach cancer [16, 17]. However, to our best knowledge, there is no specific exploration about GINS with sarcoma.

## RESULTS

### Transcriptional levels of GINS members in sarcoma patients

**Figure 1:**
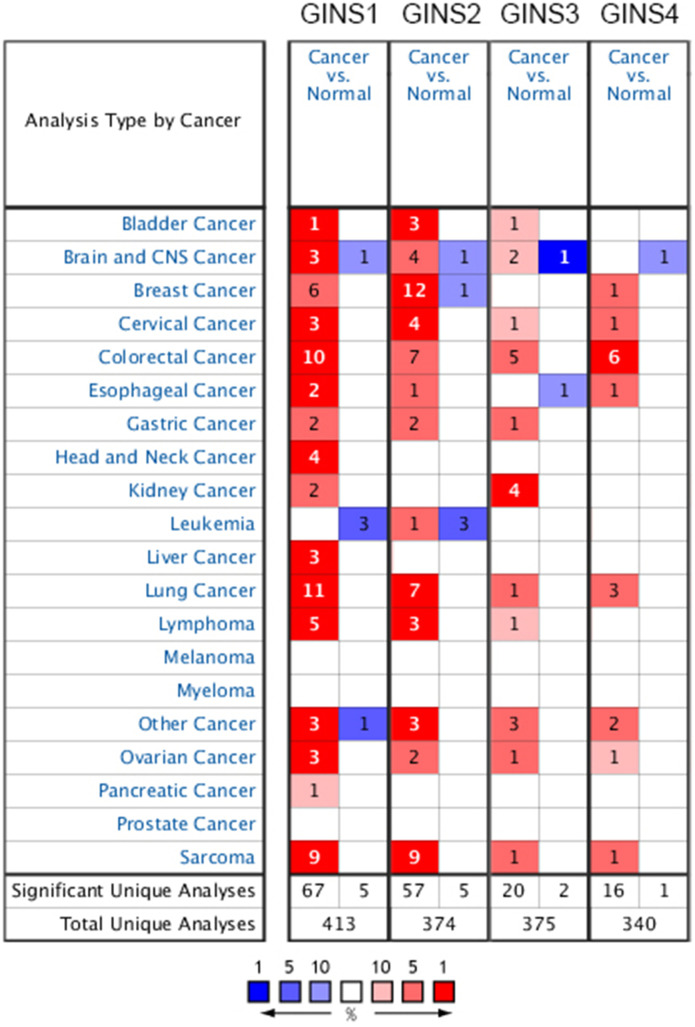
Transcription levels of GINS family genes in different tumors.

Four members (GINS1, GINS2, GINS3, GINS4) of GINS family has been discovered in previous studies. They were found to be linked to many different types of tumor. The ONCOMINE database was used to compare the dissimilarity of transcription levels of GINS between cancer and normal samples. We detected that the transcription levels of GINS were upregulated in several cancers including cervical cancer, colorectal cancer, lung cancer and sarcoma. Especially, all the transcription levels of the four subunits of GINS were upregulated in sarcoma tissues (Figure 1). In table 1, as we showed, the mRNA expression level of GINS1 was significantly upregulated in sarcoma patients. In Detwiller Sarcoma dataset [18], the mRNA expression of GINS1 in pleomorphic liposarcoma was higher than in normal samples, with a fold change of 10.222. In fibrosarcoma, malignant fibrous histiocytoma and round cell liposarcoma, GINS1 was also more highly expressed than in normal samples, and the fold changes were 9.773, 10.455 and 6.478. The Barretina Sarcoma dataset [19] provided us that the expression levels of GINS1 were higher in pleomorphic liposarcoma (fold change = 4.135), myxofibrosarcoma (fold change = 5.272), leiomyosarcoma (fold change = 4.566), dedifferentiated liposarcoma (fold change = 3.204) and myxoid/round cell liposarcoma (fold change = 3.412) than in normal samples.

**Table 1:** The significant changes of GINS expression in transcription level between different types of sarcoma (ONCOMINE database).

**Gene ID**	**Types of sarcoma vs. normal**	**Fold change**	**p value**	**t test**	**References**
GINS1	Pleomorphic Liposarcoma vs. Normal	10.222	3.37E-8	10.070	Detwiller Sarcoma [18]
	Fibrosarcoma vs. Normal	9.773	7.26E-8	7.950	Detwiller Sarcoma [18]
	Malignant Fibrous Histiocytoma vs. Normal	10.455	9.34E-8	7.561	Detwiller Sarcoma [18]
	Round Cell Liposarcoma vs. Normal	6.478	5.78E-6	6.584	Detwiller Sarcoma [18]
	Pleomorphic Liposarcoma vs. Normal	4.135	5.58E-13	12.570	Barretina Sarcoma [19]
	Myxofibrosarcoma vs. Normal	5.272	7.25E-15	16.125	Barretina Sarcoma [19]
	Leiomyosarcoma vs. Normal	4.566	4.19E-12	10.265	Barretina Sarcoma [19]
	Dedifferentiated Liposarcoma vs. Normal	3.204	6.43E-12	11.224	Barretina Sarcoma [19]
	Myxoid/Round Cell Liposarcoma vs. Normal	3.412	2.17E-11	11.565	Barretina Sarcoma [19]
GINS2	Pleomorphic Liposarcoma vs. Normal	7.103	6.10E-6	6.313	Detwiller Sarcoma [18]
	Malignant Fibrous Histiocytoma vs. Normal	6.800	7.32E-6	5.718	Detwiller Sarcoma [18]
	Fibrosarcoma vs. Normal	6.258	4.22E-5	4.924	Detwiller Sarcoma [18]
	Round Cell Liposarcoma vs. Normal	4.712	7.39E-5	4.941	Detwiller Sarcoma [18]
	Pleomorphic Liposarcoma vs. Normal	2.231	6.20E-8	7.365	Barretina Sarcoma [19]
	Myxofibrosarcoma vs. Normal	2.729	5.25E-10	9.454	Barretina Sarcoma [19]
	Leiomyosarcoma vs. Normal	2.892	2.69E-9	7.876	Barretina Sarcoma [19]
	Dedifferentiated Liposarcoma vs. Normal	2.046	2.25E-7	7.574	Barretina Sarcoma [19]
	Myxoid/Round Cell Liposarcoma vs. Normal	2.351	2.91E-8	9.018	Barretina Sarcoma [19]
GINS3	Malignant Fibrous Histiocytoma vs. Normal	5.246	3.05E-5	4.997	Detwiller Sarcoma [18]
GINS4	Fibrosarcoma vs. Normal	2.441	4.14E-5	4.971	Detwiller Sarcoma [18]

We could also learn from the Detwiller et al. [18] that GINS2 was overexpressed in pleomorphic liposarcoma, malignant fibrous histiocytoma, fibrosarcoma, round cell liposarcoma with fold changes of 7.103, 6.800, 6.258 and 4.712 respectively. Highly expressed GINS2 was also found in Pleomorphic Liposarcoma, Myxofibrosarcoma, Leiomyosarcoma, Dedifferentiated Liposarcoma, Myxoid/Round Cell Liposarcoma, with fold changes of 2.231, 2.729, 2.892, 2.046 and 2.351 respectively, compared with normal tissues through Barretina Sarcoma dataset [19]. GINS3 was found upregulated with a fold change of 5.246 in Malignant Fibrous Histiocytoma in Detwiller datasets [18]. In Detwiller datasets [18], highly overexpressed GINS4 was reported in Fibrosarcoma (fold change = 2.441).

### Comparison of the expression of GINS mRNAs in sarcoma and normal tissues

**Figure 2:**
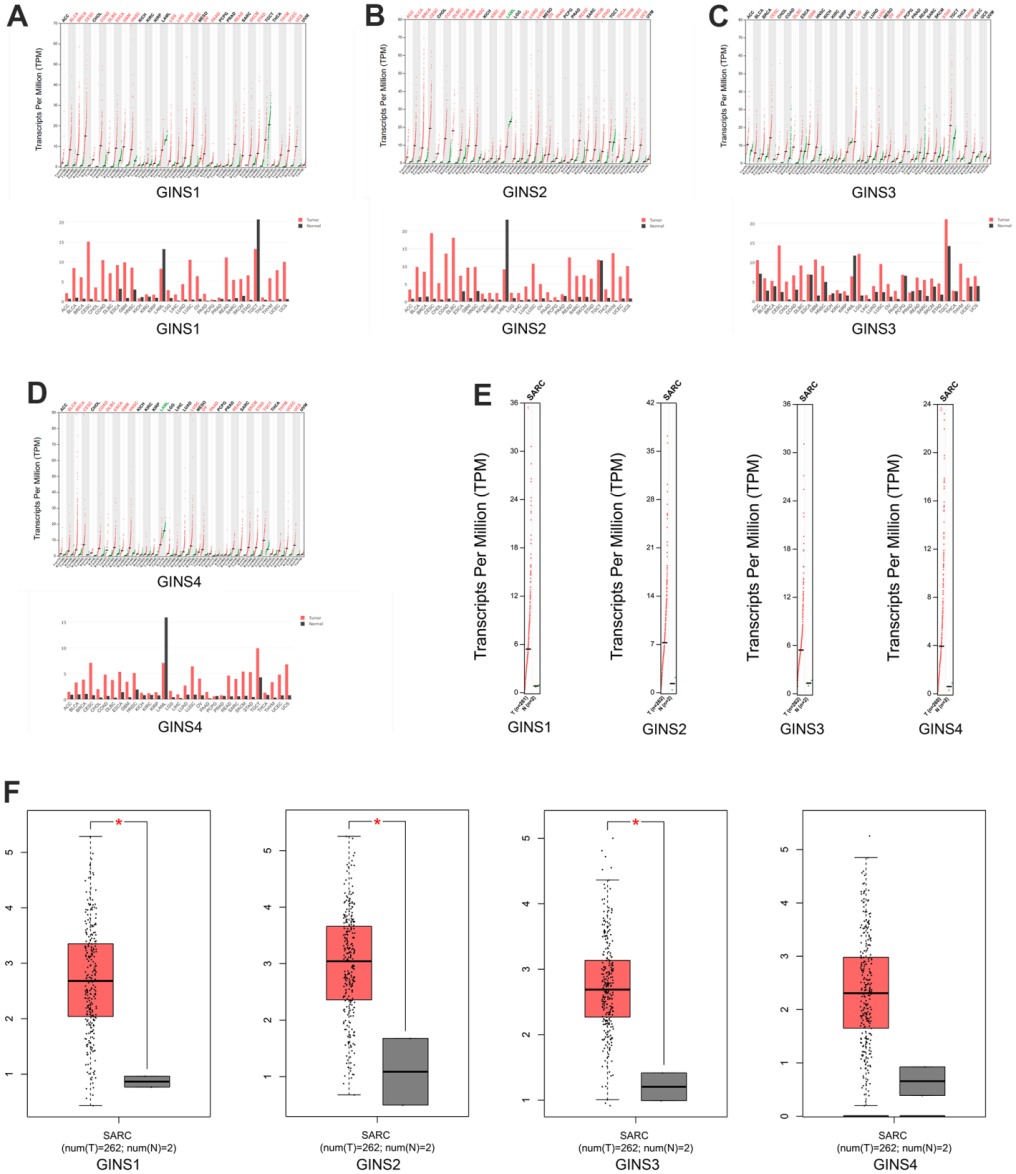
**The expression of GINS mRNA levels in sarcoma and normal tissues.** (**A**) The expression of GINS1 in tumor tissues. (**B**) The expression of GINS2 in tumor tissues. (**C**) The expression of GINS3 in tumor tissues. (**D**) The expression of GINS4 in tumor tissues. (**E**, **F**) The expression of GINS family genes in sarcoma tissue.

Using the GEPIA dataset, we compared the expression of GINS mRNAs in sarcoma and normal tissues, it indicated that the expression levels of GINS1, GINS2 and GINS3 in sarcoma were statistically higher than in normal samples. The expression level of GINS4 in sarcoma was also higher than in normal samples, but with no significance. (Figure 2A–2F).

### GINS translational factors expression in sarcoma cell lines

**Figure 3:**
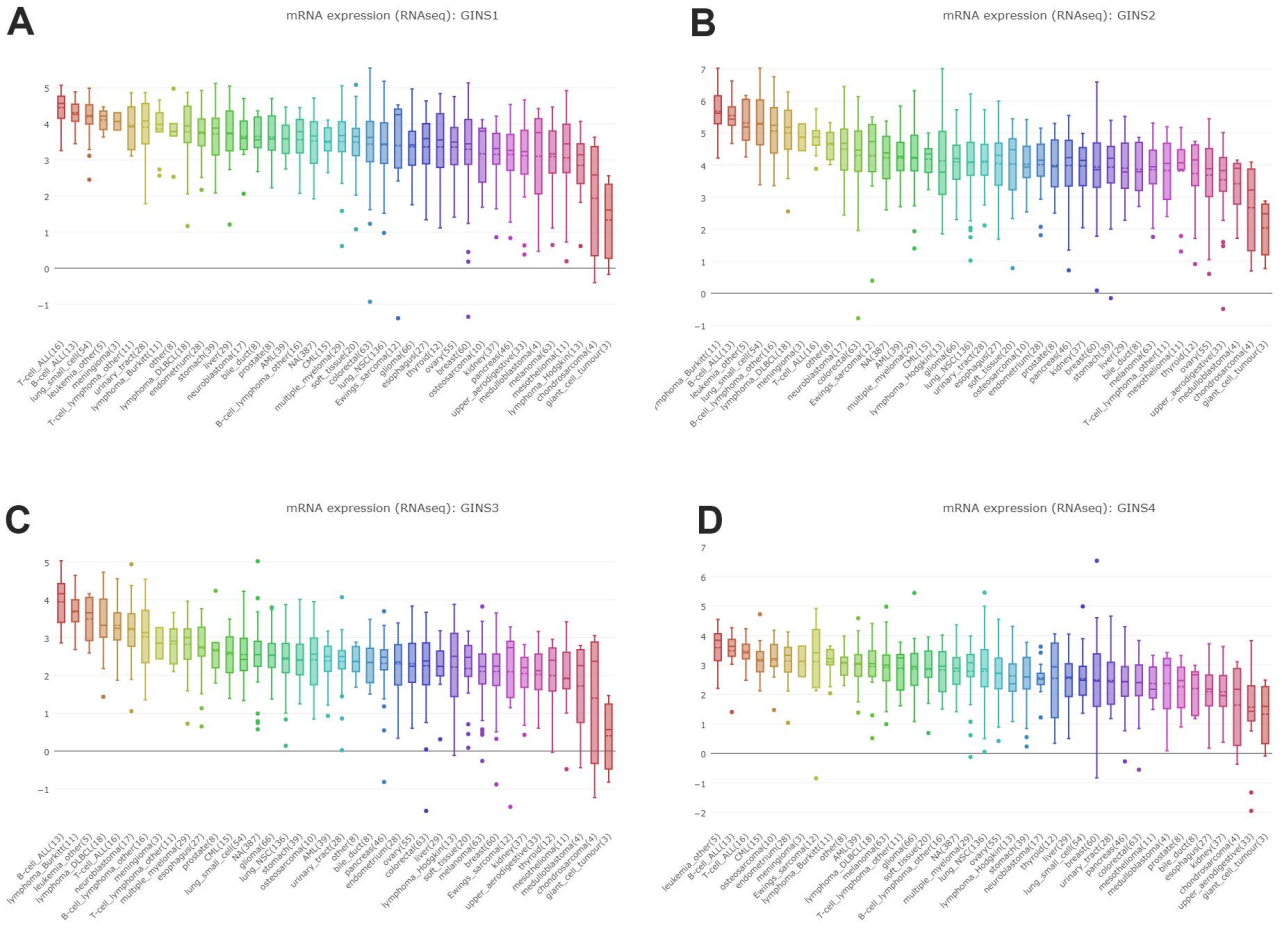
**Expression of GINS transcription factors in sarcoma cell lines.** (**A**) The expression of GINS1 in sarcoma cell lines. (**B**) The expression of GINS2 in sarcoma cell lines. (**C**) The expression of GINS3 in sarcoma cell lines. (**D**) The expression of GINS4 in sarcoma cell lines.

By interrogating CCLE, we detected the transcription level of GINS members in many types of cancer cell lines, and got the conclusion that GINS1, GINS2 GINS3 and GINS4 were highly expressed in sarcoma cell lines including Ewing's sarcoma, osteosarcoma and chondrosarcoma. (Figure 3A–3D).

### The prognostic value of GINS members in sarcoma

**Figure 4:**
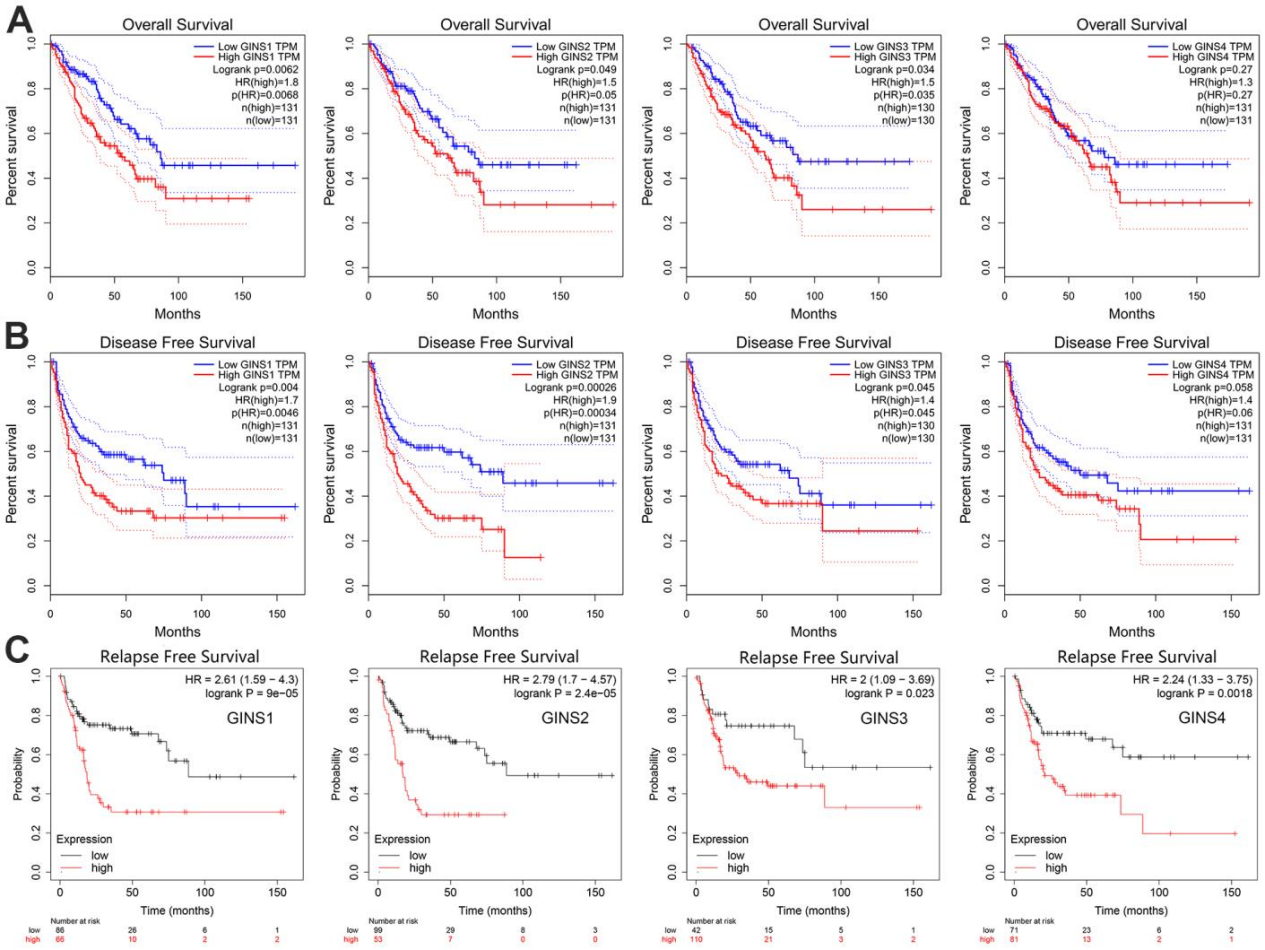
**The prognostic significance of each member of the GINS gene family in human sarcoma.** (**A**) Overall survival, (**B**) Disease-free survival, (**C**) Relapse-free survival.

The GEPIA and Kaplan-Meier Plotter was used to make the investigation of the prognostic ability of GINS1, GINS2, GINS3 and GINS4 expression levels in sarcoma. We can see from the (Figure 4A–4C), increased GINS1-3 were found to be linked to poor overall survival (OS) (p<0.05), disease-free survival (DFS) (p<0.05) and relapse-free survival (RFS) (p<0.05). Nevertheless, increased GINS4 was discovered tend to have significant association with poor RFS. To sum up, GINS1-4 were prognostic factors for sarcoma.

### Co-expressed genes of GINS genes and association between GINS genes

**Figure 5:**
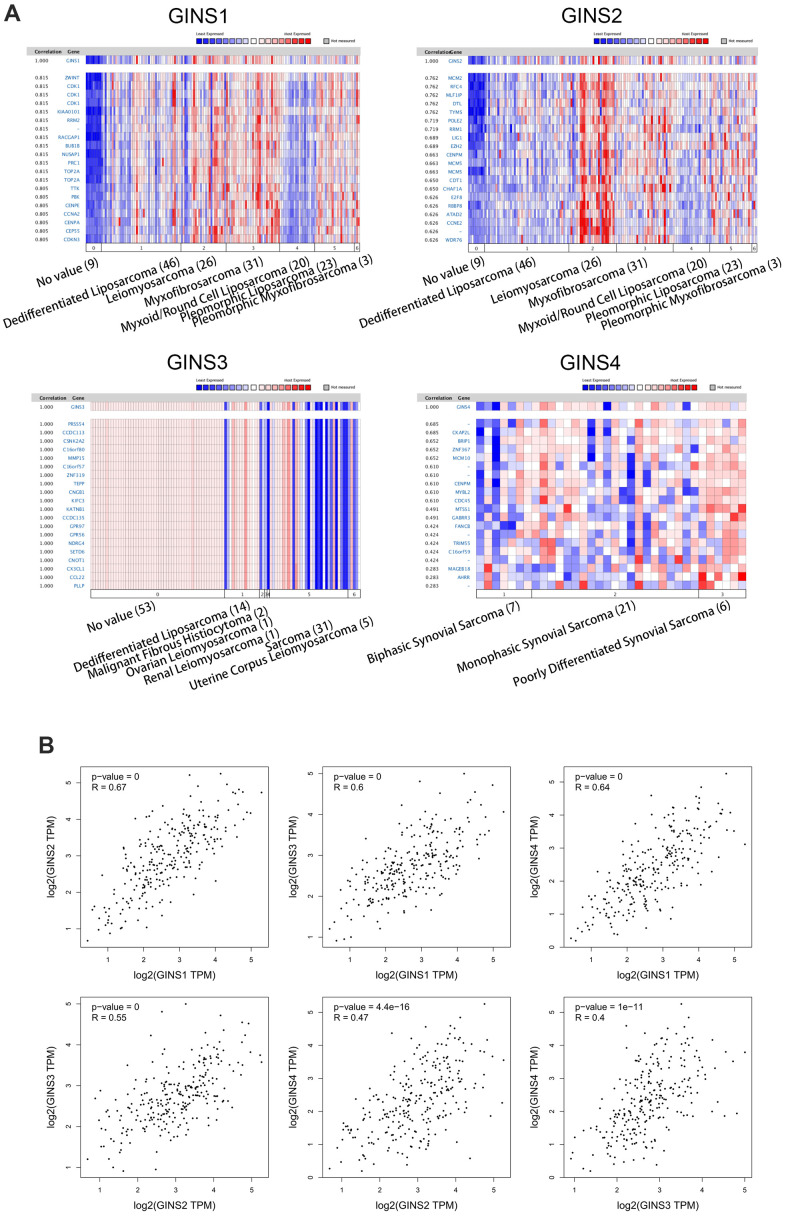
**Genes co-expressed with the GINS gene family in human sarcoma tissues.** (**A**) Co-expressed genes of the GINS gene family in sarcomas. (**B**) Correlation between members of the GINS gene family in tumors analyzed by GEPIA.

ONCOMINE and GEPIA datasets were used in this part. Analyses of co-expressed genes of GINS1 and the association between GINS1 in sarcoma were conducted in the Barretina dataset [19]. We obtained that GINS1 was highly correlated with ZWINT, CDK1, KIAA0101, RRM2, RACGAP1, BUB1B, NUSAP1, PRC1, TOP2A and TTK (Figure 5A). We searched for this of GINS2 in Barretina datasets [19] likewise, and found that it was positively related to MCM2, RFC4, MLF1IP, DTL, TYMS, POLE2, RRM1, LIG1, EZH2 and CENPM (Figure 5A). The data about co-expressed genes of GINS3 were from the TCGA Sarcoma (http://tcga-data.nci.nih.gov/tcga/), our study pointed out that GINS3 was positively correlated with PRSSS4, CCDC113, CSNK2A2, C16orf80, MMP15, C16orf57, ZNF319, TEPP, CNC81 and KIFC3 (Figure 5A). As for GINS4, we discovered it was positively correlated with CKAP2L, BRIP1, ZNF367, MCM10, CENPM, MYBL2, CDC45, MTSS1, GABRR3 and FANCB in Nakayama dataset [20] (Figure 5A). Our study also encompassed the association among GINS1, GINS2, GINS3 and GINS4 through GEPIA, revealing that GINS1 was positively correlated with GINS2 (R=0.67, p<0.05), GINS3 (R=0.6, p<0.05), GINS4 (R=0.64, p<0.05) (Figure 5B). And GINS2 was positively correlated with GINS3 (R=0.55, p<0.05) and GINS4 (R=047, p<0.05) (Figure 5B), also, GINS3 was positively correlated with GINS4 (R=0.4, p<0.05) (Figure 5B).

### Relationship between the expression level of GINS family and immune cell infiltration

**Figure 6:**
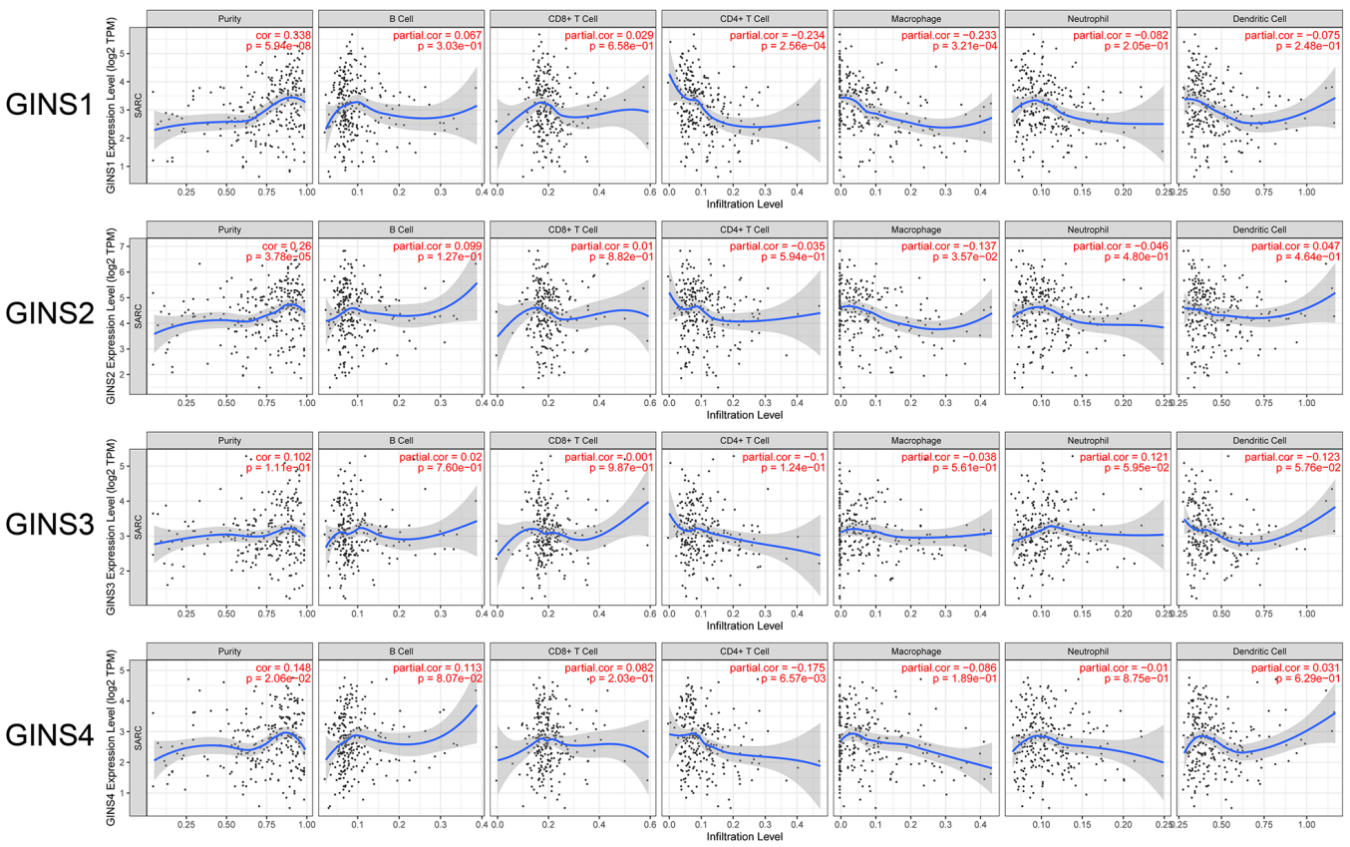
Association between GINS family genes and immune infiltration in sarcoma.

In our study, TIMER database was used to analyse the expression level of GINS family and the infiltration level of immune cells. The results showed that highly expressed GINS1 was significantly associated with the low infiltration level of CD4+ T cell (Correlation coefficient (cor) = -0.234, p<0.05) and macrophage (cor=-0.233, p<0.05). High expression level of GINS2 was significantly associated with low macrophage infiltration level (cor = -0.137, p<0.05). High GINS4 expression level was significantly correlated with low infiltration level of CD4+ T cell (cor = -0.175, p<0.05) (Figure 6).

## DISCUSSION

Sarcoma was a series of rare solid tumors and prognosis of sarcoma was poor. Sarcoma has a high degree of malignancy and the survival of patients with sarcoma remains poor. Therefore, it’s urgently needed to find the early prognostic biomarkers for sarcoma. In this study, we observed that GINS family members were all the prognostic biomarkers for sarcoma and GINS family genes could reduce the level of immune cell infiltration in the sarcoma microenvironment. All these results suggesting that GINS family genes may be potential biomarker for prognosis of sarcoma, which may be helpful for development of early prognostic biomarkers for sarcoma.

Bioinformatics analysis was a discipline using the methods of applied mathematics, informatics, statistics and computer science to study biology [21], mainly for biological mysteries of large and complex biological data [22]. Accordingly, we conducted our study using bioinformatics analysis. Previous study has reported the functional role of several GINS member in sarcoma [23–25]. This study used a bioinformatics approach to investigate the expression of the GINS family in sarcoma and its impact on the prognosis of sarcoma patients, and to further investigate the relationship with immune cell infiltration in the sarcoma microenvironment. Furthermore, we probed into the correlation of all GINS family in sarcoma and its co-expressed genes. Dense-isotope substitution experiments discovered that GINS complex is important element rather than a passing traveler in initiation and process of replication [26]. Due to our research, a conclusion can be drawn that all the four subunits of GINS are overexpressed in sarcoma tissues, compared with normal tissues. The four GINS family members could make contributions to the treatment to sarcoma. The high expression level of GINS1, GINS2 and GINS4 could reduce immune cell infiltration in the sarcoma microenvironment. Moreover, expression of all the four members encoding the subunits for GINS complex positively correlated with each other.

Due to previous study, Stable association of MCM complex and GINS complex form the CMG complex, which is responsible for the activity of DNA helicases [27]. The increased expression of GINS family members was involved in many different types of cancers and procedures [8–17]. It was associated with worse prognosis. Tang et al. discovered that the inhibition of GINS1 expression significantly weakened the proliferation *in vitro*, but accelerated the apoptosis of deviant cells [28]. Therefore, they defined it as a target of osteosarcoma. We’ve described about that in mice experiment, knockdown of GINS1 expression caused inhibition of tumor growth [29]. On the contrary, overexpressed levels of GINS1 lead to high proliferation [30]. Through the research by Bu et al., the levels of GINS1 also in connected with the development of pancreatic cancer [31]. This is also confirmed by the research of Fu et al. [32]. Our study indicated that the value of GINS1 in prognosis of human sarcoma was confirmed by 131 sarcoma patients. With the increased expression levels, there are also significant differences in DFS and OS.

GINS2 also found to be associated with tumors, by regulating PI3K/Akt and MEK/ERK signaling pathways, GINS2 facilitated cell proliferation, migration, invasion, and EMT in NSCLC [33]. Tian et al. also determined the identification of GINS2 as the hub gene though the WGCNA and PPI network. GINS2 was also reported to take part in DNA replication and cell progression as a companion of Sld five 2 [34]. And another pathway has been found in non-small-cell lung cancer by Chi et al., it indicated that via p53/GADD45A pathway, GINS2 knockdown down-regulated cell proliferation, causes G2/M phase cell cycle arrest and augments apoptosis conversely. In human sarcoma, our results revealed that the expression level of GINS2 in sarcoma tissues was higher than it in normal tissues. By GEPIA dataset, we confirmed the prognosis value of GINS2, its increased expression was linked to poor DFS and OS.

As for GINS3, the expression level of Psf3, which is encoded by GIINS3, in the study of Tauchi et al. in 2016 [35], was found to be association with age, gender, T factor, lymph node metastasis, stage and P factor in lung adenocarcinoma. Besides, poor DFS and OS were matched by higher expression of it [35]. After 3 years, they confirmed that Psf3 is the independent prognostic factor for lung adenocarcinoma through further discoveries by multivariate analysis. Additionally, they revealed that in the patients, who received postoperative chemotherapy, with high expression of Psf3 in stage 1, the five-year survival rate was much greater. Conversely, the same result was not been observed in patients with low expression, which signified the Psf3 may serve as a biomarker of adjuvant tegafur-uracil administration to treat lung adenocarcinoma in stage 1 [36]. In another Japanese research, Psf3, as a member of CMG complex, accelerated excessive proliferation also in non-small-cell lung cancer [37]. These are consistent with our study, in human sarcoma, GINS3 may be a potential biomarker and its expression have influence on the prognosis.

GINS4 was also reported expressed higher in gastric cancer, non-small-cell lung cancer, colorectal cancer and breast cancer than normal tissues [16, 17, 38, 39]. There were different pathways, for example, in gastric cancer, via directly combination with Rac1/CDC42, GINS4 activated Rac1/CDC42 and then influence the downstream pathways [16]. In colorectal cancer, the KLF/GINS4 might be the crucial factor [17]. Our study found high expression of GINS4 in human sarcoma tissues, and it tended to have tight connection with OS and DFS, but failed to achieve significance.

The tumor microenvironment has a very important impact on the progression and recurrence of many types of cancer. Previous studies have demonstrated that immune cells in the tumor microenvironment can promote or suppress cancer activity and have been used as an important determinant of immunotherapy efficacy and clinical prognosis. In the present study, we found that the expression level of GINS gene was significantly correlated with the level of immune cell infiltration, suggesting that GINS gene can be used to assess the immune status of sarcoma. Our study provides additional evaluation indicators for the effectiveness of immunotherapy in sarcoma patients.

The efficiency and convenience of bioinformatics are obvious, the datasets we used helped us to unearth the expression and prognosis of GINS family in human sarcoma. There are also limitations of our study, we used almost every practicable data, it still seems to be inadequate, more future studies and more datasets are needed. We didn’t find the pathways of GINS family in human sarcoma, and we will keep on and achieve Immunohistochemical experiments.

## CONCLUSIONS

In conclusion, we observed that GINS family members were all highly expressed in sarcoma tissue and high expression of GINS members in sarcoma was closely related to sarcoma tumorigenesis. The prognostic relevance tested was also obvious, increased expression of GINS1, GINS2, GINS3 and GINS4 may provide us new therapeutic targets for the treatment of sarcoma. GINS1, GINS2 and GINS4 were positively correlated with the immune cell infiltration in sarcoma microenvironment. All the GINS members could be seen as potential prognostic and immune markers to improve prognostic accuracy of sarcoma.

## MATERIALS AND METHODS

### ONCOMINE analysis

The online data-mining platform and cancer microarray database ONCOMINE (https://www.oncomine.com/) was used to analyze the transcription levels of GINS members among different cancers [40]. P value was generated by the Student t-test, the cut-off of p-value and fold-change were defined as 0.05 and 2, respectively. and the mRNA expressions of the GINS family subunits in cancer samples was compared with normal ones. We also analyzed genes correlated with GINS members in sarcoma by using ONCOMINE [41].

### GEPIA analysis

Gene Expression Profiling Interactive Analysis (GEPIA) (http://gepia.cancer-pku.cn/) is a newly developed multi-function data analysis platform based on the Cancer Genome Atlas (TCGA) and the Genotype-Tissue Expression (GTEx). GEPIA includes 9736 tumors and 8587 normal samples. It was used to compare the different expression levels of GINS members between the human sarcomas and the normal samples. It also provided us survival analysis of sarcoma patients, including OS and DFS, the patients were divided into two groups according to the median expression of GINS (high and low expression). We also calculated log-rank P-value and the cox proportional hazard ratio (HR) with 95% confidence intervals [42, 43].

### Kaplan-Meier plotter

Kaplan-Meier Plotter (http://www.kmplot.com) is an online database which includes the expression levels of different genes and their prognostic values in pan-cancer. In our study, it was used to analyze the different relapse-free survival (RFS) of sarcoma patients with high GINS expression level and low GINS expression level. The hazard ratio (HR) with 95% confidence interval and the lop-rank P-value were also calculated [44].

### CCLE dataset

Cancer Cell Line Encyclopedia (CCLE) (https://portals.broadinstitute.org/ccle) was established to deeply and accurately depict the genetic characteristics of cancer. It was between the Broad Institute and the Novartis Institutes for Biomedical Research and its Genomics Institute of the Novartis Research Foundation. CCLE conducted a large-scale in-depth sequencing of 947 human cancer cell lines covering more than 30 tissue sources, integrating genetic information such as DNA mutations, gene expression and chromosome copy numbers. The CCLE was an access to provide us the genomic data, visualization cell lines. The GINS family’s expression in human sarcoma cell lines was obtained from the CCLE dataset [45].

### TIMER dataset analysis

The TIMER database (https://cistrome.shinyapps.io/timer/) is a comprehensive resource for systematical analysis of immune infiltrates across diverse cancer types. In our study, it was used to analyze the correlation between the expression level of GINS family and infiltration level of immune cells.

### Availability of data and materials

The datasets used and/or analyzed for this study were obtained from the ONCOMINE (https://www.oncomine.com/), Gene Expression Profiling Interactive Analysis (GEPIA) (http://gepia.cancer-pku.cn/), Kaplan-Meier Plotter (www.kmplot.com) and Cancer Cell Line Encyclopedia (CCLE) (https://portals.broadinstitute.org/ccle).
